# Adult population as potential reservoir of NTD infections in rural villages of Kwale district, Coastal Kenya: implications for preventive chemotherapy interventions policy

**DOI:** 10.1186/1756-3305-4-175

**Published:** 2011-09-14

**Authors:** Sammy M Njenga, Charles S Mwandawiro, Erastus Muniu, Mariam T Mwanje, Fatma M Haji, Moses J Bockarie

**Affiliations:** 1 Eastern and Southern Africa Centre of International Parasite Control (ESACIPAC), Kenya Medical Research Institute (KEMRI), Mbagathi Road, Nairobi, Kenya; 2 Centre for Public Health Research (CPHR), Kenya Medical Research Institute (KEMRI), Mbagathi Road, Nairobi, Kenya; 3 Division of Vector-Borne & Neglected Tropical Diseases (DVBNTD), Ministry of Public Health & Sanitation, Nairobi, Kenya; 4 Kwale District Hospital, Kwale County, Coast Region, Kenya; 5 Centre for Neglected Tropical Diseases, Liverpool School of Tropical Medicine, Liverpool, UK

## Abstract

**Background:**

Neglected tropical diseases (NTDs) are major public health problems in developing countries where they contribute to suffering of populations living in poor settings. As part of a research project started in September 2009 in Kwale district, Coast Region, Kenya, a baseline cross-sectional survey was conducted in 5 rural villages to provide information on the status of NTDs, including urinary schistosomiasis, soil-transmitted helminthiasis (STH), and lymphatic filariasis. This paper presents the results of a parasitological investigation among adults in the study villages.

**Methods:**

A total of 599 adults in the 5 study villages were tested for NTD infections in urine, stool and blood. The presence of *Schistosoma haematobium *infection was determined by the urine filtration method. The presence of STH in stool was determined by Kato-Katz method while filarial antigenaemia was determined using immunochromatographic (ICT) test.

**Results:**

The study revealed high prevalence of hookworm (41.7%) and schistosomiasis (18.2%) infections among adults in the study villages. Of the 599 individuals examined, 50.1% had one or more helminthic infections. There was low level of polyparasitism with helminthic NTDs in the study population with 9.5% and 1.7% of the participants having two and three infections, respectively.

**Conclusions:**

In the current study, hookworm and schistosomiasis infections were identified as important infections among adults living in areas of high endemicity for these infections. Thus, if this section of the population is left untreated it may remain an important potential reservoir and a source of re-infection for school-age children treated in school deworming programmes. Therefore, there is a need to design novel strategies for preventive chemotherapy interventions that could allow inclusion of adults in an effort to reduce force of infection in high endemic communities.

## Background

Neglected tropical diseases (NTDs) include seven helminth infections namely soil-transmitted helminths (STH) infections (hookworm, ascariasis, trichuriasis), lymphatic filariasis (LF), onchocerciasis, guinea worm disease (dracunculiasis), and schistosomiasis [1]. Soil-transmitted helminths (STH) and schistosomiasis are mostly prevalent in developing countries due to poor sanitation and lack of adequate clean water. Soil-transmitted helminth infections are among the most prevalent infections in the world with around 1.4 billion individuals infected by *Ascaris lumbricoides*, 1.0 billion by *Trichuris trichiura*, and 1.3 billion by hookworms [2]. Of an estimated 200 million people infected with schistosomiasis, 170 million live in sub-Saharan Africa while the remaining 30 million live in North Africa, Asia and South America [3]. Lymphatic filariasis is a major cause of debilitating chronic disability and is estimated to infect over 120 million people worldwide [4]. The coastal areas of Kenya are highly endemic for urinary schistosomiasis, STH, and LF [5-7]. Children have the highest prevalence and intensity of STH and schistosomiasis infections, but the consequences of chronic infection, such as growth stunting, anaemia, hepatic/urinary fibrosis, and impaired cognitive development, continue to have an effect throughout adulthood [8].

For a long time most national health authorities in sub-Saharan Africa have shown little commitment to control of NTDs mainly due to lack of resources [9]. However, the situation is now changing with the realization that deworming is a relatively simple, effective, and cost-effective public health measure that can be readily integrated with existing health care programmes. This has led to renewed interest in control of parasitic diseases; in 1997, the 50^th ^World Health Assembly (WHA) passed a resolution to eliminate LF globally as a public health problem [10]. Following the WHA resolution on LF elimination, the World Health Organization (WHO) launched the Global Programme to Eliminate Lymphatic Filariasis (GPELF) in 2000 [11]. Subsequently in 2001, during the 54^th ^WHA, the WHO urged its member states to ensure provision for the regular anthelminthic treatment of all school-age children living in areas of schistosomiasis and STH endemicity [2]. Targeting anthelminthics to school-age children capitalizes on the fact that the heaviest burdens of infection are found in this segment of the population. In 2009, a research project to evaluate an alternative strategy for controlling morbidity due to schistosomiasis and STH and its integration with the national LF elimination programme was established in Kwale district, Coast Region, Kenya. The current study was conducted to assess the levels of schistosomiasis, STH and LF infections in adults living in 5 rural villages selected for the research project.

## Methods

### Study site and design

This cross-sectional study was conducted in January 2010 in 5 villages in Mwaluphamba location, Kwale district, Coast Region, Kenya. Kwale district is in Kwale County and covers an area of 1,043 km^2 ^which is divided into two administrative divisions, namely Matuga and Kubo divisions: the study area is situated in Kubo division. The populations of Kwale County and Kwale district based on 2009 census were estimated to be 649,931 and 151,978 persons, respectively. In terms of climate, the district is hot and dry from January to March and relatively cool from June to August. Rainfall pattern is bimodal with long rains normally occurring between mid-March and June while the short rains occur between October and December.

Subsistence farming of food crops including maize, cowpeas and cassava is done mainly for domestic consumption. Coconuts, oranges, mangoes and cashew nuts are also grown for both domestic consumption and as cash crops. The main livestock kept include cattle, goats, ducks and chickens. The protocol for this study was reviewed and approved by the Scientific Steering and Ethical Review Committees of the Kenya Medical Research Institute (KEMRI). Oral informed consent was obtained from study participants.

### Sample collection and examination

Convenience samples of approximately 150 adults in each village were recruited to participate in the study on a voluntary basis. Stool containers were given to study participants the day before sample collection. Collection of samples was done the following morning at designated places (usually schools) agreed at meetings held in each village, but there were some people who did not turn up for sample collection. Samples collected included stool, urine, and 3 ml venous blood. The samples were transported to KEMRI field station laboratory located at the Kwale district hospital for processing.

All samples were obtained between 10:00 am. and 1:00 pm. Urine samples were collected in wide-mouth plastic containers. Immediately the urine samples were collected, 10 ml specimens were used for examination of schistosome eggs using urine filtration technique. Briefly, the 10 ml urine specimens were aliquoted using disposal syringes and filtered through 12 μm polycarbonate membrane filters at the sample collection site. The filters were carefully placed on labeled microscope glass slides and stored in slide boxes for later reading under a light microscope. Additional 5 ml aliquots of urine specimens preserved with sodium azide (0.1% w/v) were collected for later assessment of morbidity due to *S. haematobium *infection.

Lymphatic filariasis infection was assessed in 100 μl venous blood aliquots using the immunochromatographic (ICT) test which detects circulating filarial antigens. Thick and thin blood smears were made on clean microscope slides and air-dried before storage in slide boxes. The thin smear was fixed with methanol and then both smears stained with 10% Giemsa stain, pH 7.0 for 10 min. The smears were rinsed briefly in PBS buffer before drying at room temperature. The thick smear was used to examine for malaria parasites under oil immersion objective and the thin film used to determine species for positive slides. The remaining blood specimens were used for preparation of plasma samples to be used later for serological assays related to the research project. The plasma and urine specimens were stored at 4°C while at the KEMRI field station and frozen at -80°C upon return to central laboratories in Nairobi.

The quantitative Kato-Katz method was used to examine the stool samples for presence of different intestinal helminth species eggs as previously described. Briefly, two 41.7 mg stool smears were made from each stool sample on the day of sample collection for examination and counting of STH eggs. The smears were read within 1 hour for hookworm eggs and next day for other STH eggs. Egg counts were expressed as eggs per gram of stool (EPG). Individuals were categorized as having light- or heavy-intensity infections based on the criteria of the WHO [12]. For *S. haematobium*, light-intensity infections were 1 - 49 eggs/10 ml urine and heavy-intensity infections ≥ 50 eggs/10 ml urine. Hookworm infections were classified as light-intensity when counts were1-1,999 EPG and heavy-intensity when counts were ≥ 2000 EPG.

### Data management and analysis

Data were double-entered into a computer using MS Access and after validation analyzed using SPPS 12.0 statistical analysis software (SPSS Inc., Chicago, Illinois, USA). Chi-square (X^2^) test was used to compare proportions between the villages. One-way Analysis of Variance (ANOVA) was used to compare differences between group means and Kruskal-Wallis one-way ANOVA to compare group medians. A value of P < 0.05 was considered statistically significant.

## Results

Demographic characteristics of the study group are summarized in Table 1. A greater number of female participants were examined compared to males (p < 0.01). Additionally, female participants were significantly younger (median age 35 years, range 15 - 88 years) compared to male participants (median age 46 years, range 18 - 85 years); (P < 0.001).

**Table 1:** Demographic characteristics of study participants in five villages, Kwale district, coastal Kenya

Characteristic	Maponda	Mirihini	Kajiweni	Miatsani	Mlafyeni	All
**No. tested**	116	125	122	93	137	593
*Female	77	94	69	70	99	409
**Male**	39	31	53	23	38	187
**Median age (yrs)**	38	33	40	41	38	38
**Age range (yrs)**	18-85	18-80	15-83	18-82	18-88	15-88

*There were more female participants and they were significantly younger compared to male participants (P < 0.001). Data on sex/age for 6 participants were missing.

Prevalence of five helminthic infections and malaria among the study villages are summarized in Table 2. There was high prevalence of hookworm (41.7%) and schistosomiasis (18.2%) infections among adults in the study villages. Of 107 persons with *S. haematobium *infection, 49 (45.8%) had hookworm infection, 2 (1.9%) had *T. trichiura*, 7 (6.7%) had LF, and 6 (5.7%) had malaria. The general prevalence of malaria in the study area was 5.6% and except for one village (Miatsani) malaria was prevalent in all other four villages.

**Table 2:** Prevalence of urinary schistosomiasis, STH, LF and malaria among study participants in five rural villages in Kwale district, coastal Kenya

	No. infected/No. examined (%) 95% CI (%)					
	
Village	Ascaris	Trichuris	Hookworm	Schisto	LF	Malaria
**Maponda**	0/106 (0) -	4/106 (3.8) 0.2-7.4	40/105 (38.1) 28.8-47.4	10/117 (8.5) 3.4-13.5	6/118 (5.1) 1.1-9.1	9/118 (7.6) 2.8-12.4
**Mirihini**	0/121 (0) -	0/122 (0) -	58/119 (48.7) 39.7-57.7	18/127 (14.2) 8.1-20.3	4/126 (3.2) 0.1-6.3	4/127 (3.1) 0.1-6.1
**Kajiweni**	2/122 (1.6) 0-3.8	1/122 (0.8) 0-2.4	64/120 (53.3) 44.4-62.2	25/122 (20.5) 13.3-27.7	7/122 (5.7) 1.6-9.8	12/120 (10.0) 4.6-15.4
**Miatsani**	0/92 (0) -	1/92 (1.1) 0-3.2	44/93 (47.3) 37.2-57.4	20/93 (21.5) 13.2-29.8	4/91 (4.4) 0.2-8.6	0/93 (0) -
**Mlafyeni**	2/129 (1.6) 0-3.8	0/129 (0) -	29/127 (22.8) 15.5-30.1	34/128 (26.6) 18.9-34.3	4/137 (2.9) 0.1-5.7	8/134 (6.0) 2.0-10.0
**All**	4/570 (0.7) 0-1.4	6/571 (1.1) 0.2-2.0	235/564 (41.7) 37.6-45.8	107/587 (18.2) 15.1-21.3	25/594 (4.2) 2.6-5.8	33/592 (5.6) 3.7-7.5
*P value			< 0.001	0.030	0.765	0.015

* Chi-square test. For *Ascaris *and *Trichuris *infections, Chi-square was not valid hence p-values not indicated

There was no significant difference in prevalence of STH and schistosomiasis infections between males and females. The prevalence of LF infection, however, was significantly higher in males (8.2%) than in females (2.2%); (p < 0.01). Intensities of hookworm and schistosomiasis infections among adults positive for these infections in the 5 study villages are summarized in Table 3. The majority of these infections were light-intensity and there were no statistically significant differences among the 5 villages. Overall, > 95% of hookworm and > 75% of schistosomiasis infections were categorized as light-intensity. Additionally, *Ascaris *and *Trichuris *infections were light-intensity.

**Table 3:** Intensities of hookworm and urinary schistosomiasis infections among adults positive for the infections in five rural villages in Kwale district, coastal Kenya

	Village						
	
Infection	Maponda	Mirihini	Kajiweni	Miatsani	Mlafyeni	All	P
**Hookworm**							
No. Positive	40	58	64	44	29	235	
GMI EPG	162	147	143	140	114	142	0.949
Light (%)	37 (92.5)	55 (94.8)	63 (98.4)	42 (95.5)	29 (100.0)	226 (96.2)	
Heavy (%)	3 (7.5)	3 (5.2)	1 (1.6)	2 (4.5)	0 (0)	9 (3.8)	
**Schistosomiasis**							
No. Positive	10	18	25	20	34	107	
GMI (eggs/10 ml urine)	8	11	11	14	28	15	0.147
Light (%)	10 (100.0)	15 (83.3)	19 (76.0)	15 (75.0)	23 (67.6)	82 (76.6)	
Heavy (%)	0 (0)	3 (16.7)	6 (24.0)	5 (25.0)	11 (32.4)	25 (23.4)	

Geometric mean intensities (GMI) and levels of the intensities (light or heavy) were computed for individuals positive the infections only

Hookworm: light, 1-1999 EPG; heavy, ≥ 2000 EPG.

*S. haematobium*: light, 1 - 49 eggs/ml urine; heavy, ≥ 50 eggs/ml urine

The status of polyparasitism in the study area is summarized in Table 4. Overall, 50.1% of the study population had one or more helminthic infections. There was low level of polyparasitism with helminthic NTDs in the study population with 9.5% and 1.7% of the participants having two and three infections, respectively.

**Table 4:** Polyparasitism with urinary schistosomiasis, soil-transmitted helminths, and lymphatic filariasis among adults in 5 rural villages in Kwale district, coastal Kenya

		No. of infections (%)			
		
Village	N	0	1	2	3
**Maponda**	118	72 (61.0)	35 (29.7)	8 (6.8)	3 (2.5)
**Mirihini**	127	60 (47.2)	54 (42.5)	13 (10.2)	0 (0)
**Kajiweni**	123	43 (35.0)	63 (51.2)	15 (12.2)	2 (1.6)
**Miatsani**	94	41 (43.6)	38 (40.4)	14 (14.9)	1 (1.1)
**Mlafyeni**	137	83 (60.6)	43 (31.4)	7 (5.1)	4 (2.9)
**All**	599	299 (49.9)	233 (38.9)	57 (9.5)	10 (1.7)

## Discussion

Few studies have been conducted to quantify the extent of schistosomiasis and STH infections among adults living in areas of high endemicity for these infections. Data from this study revealed a relatively high prevalence of *S. haematobium *and hookworm infections in the adult population. Current WHO guidelines recommend preventive chemotherapy based on regular anthelminthic drugs as a public health intervention to control helminthic infections and reduce morbidity [13]. Many public health programmes targeting STH and schistosomiasis currently employ school-based deworming which has been previously shown to provide externality benefits to untreated groups within and close to the treatment schools [14]. The results of our study, however, suggest that if adults are left untreated in areas where prevalence of helminthic infections is high in this group, the adults may act as a reservoir for transmission and a source of reinfections to the school-age children and thus present an obstacle to the control programmes.

On average, around 4% of the adults in this study had circulating filarial antigen (CFA). By the time this study was conducted, Kwale district had received three rounds of diethylcarbamazine citrate (DEC)/albendazole mass drug administration (MDA) against LF (in September 2003, March 2005, and December 2008) under the National Programme for Elimination of LF (NPELF). A previous LF research study by our team in Malindi district in the northern area of Coast Region, reported that prevalence of CFA had declined from approximately 34% to 11% after 4 rounds of MDA [15]. The results of this study suggest that LF, compared to schistosomiasis and STH, is not a major public health problem in the study area. However, continued MDA for LF is still recommended in this setting and all other endemic areas to meet the WHO target of global elimination of the disease as a public health problem.

Other studies have shown that DEC and albendazole co-administration during LF MDA has ancillary health benefits of also controlling STH infections [16,17]. It is likely that any additional health gains in controlling STH made during the 3 rounds of DEC/albendazole MDA administered in Kwale district were lost when the MDAs were missed for long periods. Reinfection with STH is a common problem especially in areas of high transmission.

Additionally, our findings show that malaria is also endemic in the area. Efforts should be made to integrate the often well-funded malaria programmes with specific NTDs occurring in different areas. It may be prudent to consider integration of malaria control and schistosomiasis/STH control in the study setting and other similar areas. Malaria programmes in sub-Saharan Africa are currently scaling up towards universal coverage for all populations at risk of malaria with locally appropriate interventions, including insecticide-treated nets (ITNs), so that all age groups can be protected against malaria. Addition of deworming against schistosomiasis and STH to ITN distribution campaigns in highly endemic areas would provide further health benefits for the local communities and may be a cost-effective way of using the available resources.

The major potential weakness of this study may be the fact that only one stool sample was collected. The accuracy of the Kato-Katz technique in identifying individuals with STH infections is limited by day-to-day variation in helminth egg excretion and sensitivity is greatly reduced when intensity of infections is low [18]. Improved detection of STH eggs in stool requires examination of stool specimens collected on 2 to 3 consecutive days which may not be practical especially when working in very remote areas. A new technique known as FLOTAC has been proposed as a better tool for diagnosis of STH infections; in hookworms infection study conducted in Cote d'Ivoire, the FLOTAC technique was found to have a sensitivity of 88.2% compared with 68.4% for Kato-Katz [19]. Thus, the prevalence of STH infections, and in particular hookworms, reported in the current study may have been higher if the more sensitive FLOTAC technique was used.

It is estimated that 26 - 68% of individuals with schistosomiasis are carriers of an additional helminthic infection, such as hookworms, *A. lumbricoides*, or *T. trichiura *[20]. This study revealed that approximately 46% of adults with schistosomiasis also had hookworms. The additional parasites may aggravate morbidity due schistosomiasis infection, especially in young children [21]. Hookworm infection is a well-known cause of iron-deficiency anaemia because of intestinal blood loss. Anaemia predisposes to severe morbidity in children and pregnant women [22]. However, the majority of schistosomiasis and hookworm infections in our study were light-intensity which suggests that the effects may be quite mild.

The WHO Expert Committee on the Control of Schistosomiasis and STH during its meeting in 2001 noted that there are substantial reductions in the cost of anthelminthic drugs generally, and said that new options for treatment and retreatment strategies should be considered [2]. Since praziquantel is now available at low cost (approximately US$0.2 per treatment) more liberalized use of the drug may become possible [23]. The results of this study suggest that it may be necessary to develop novel approaches to offer preventive chemotherapy interventions to adults in high endemic areas in an effort complement school-based deworming and to accelerate reduction of transmission of helminthic infections in such areas. Inclusion of adults in control programmes may have the potential to dramatically improve both efficiency and effectiveness of control efforts against NTDs employing integrated MDAs. It may be useful to start pilot NTDs integrated control programmes in endemic areas which may later be scaled up to regional or national programmes.

## Conclusions

The findings of this study underscore schistosomiasis and STH infections as important infections among adults in areas of high endemicity for these infections. Additionally, these results suggest need for design of optimal treatment approaches that may consider including adults at risk of these infections in preventive chemotherapy interventions. Reduction of these infections in adult populations may contribute to subsequent decrease in the force of transmission in such communities leading to lower morbidities in children.

## Competing interests

The authors declare that they have no competing interests.

## Authors' contributions

SMN participated in the conception and design of the study, data collection and analyses, and drafted the manuscript. SMN, MTM and FMH supervised specimen collection and laboratory examinations. CSM participated in the conception and design of the study. SMN drafted the manuscript, whereas MJB and MTM revised it critically for intellectual content. EM helped in data management and analysis of the data. All authors read and approved the final manuscript. SMN is the guarantor of the paper.

## References

[B1] Hotez PJ Molyneux DH Fenwick A Ottesen E Ehrlich Sachs S Sachs JD Incorporating a rapid-impact package for neglected tropical diseases with programs for HIV/AIDS, tuberculosis, and malaria PLoS Med 2006 3 5 e102 10.1371/journal.pmed.0030102 16435908 PMC1351920

[B2] World Health Organization Prevention and control of schistosomiasis and soil-transmitted helminthiasis: report of a WHO expert committee WHO Technical Report Series No 912 2002 Geneva 12592987

[B3] World Health Organization Programme Report no. 17 - Seventeenth Programme Report of the UNICEF/UNDP/World Bank/WHO Special Programme for Research & Training in Tropical Diseases 2005 Geneva

[B4] Michael E Bundy DA Grenfell BT Re-assessing the global prevalence and distribution of lymphatic filariasis Parasitology 1996 112 Pt 4 409 428 8935952 10.1017/s0031182000066646

[B5] Muchiri EM Ouma JH King CH Dynamics and control of Schistosoma haematobium transmission in Kenya: an overview of the Msambweni Project Am J Trop Med Hyg 1996 55 5 Suppl 127 134 8940966 10.4269/ajtmh.1996.55.127

[B6] Brooker S Peshu N Warn PA Mosobo M Guyatt HL Marsh K Snow RW The epidemiology of hookworm infection and its contribution to anaemia among pre-school children on the Kenyan coast Trans R Soc Trop Med Hyg 1999 93 3 240 246 10.1016/S0035-9203(99)90007-X 10492749

[B7] Njenga SM Wamae CN Mwandawiro CS Molyneux DH Immuno-parasitological assessment of bancroftian filariasis in a highly endemic area along the River Sabaki, in Malindi district, Kenya Ann Trop Med Parasitol 2007 101 2 161 172 10.1179/136485907X156933 17316502

[B8] Wiest PM The epidemiology of morbidity of schistosomiasis Parasitol Today 1996 12 6 215 220 10.1016/0169-4758(96)10019-3 15275200

[B9] Engels D Chitsulo L Crompton D, Montresor A, Nesheim M, Savioli L Schistosomiasis Controlling disease due to helminth infections 2003 Geneva

[B10] World Health Organization Elimination of lymphatic filariaisis as a public health problem 1997 Geneva WHA50/1997/REC/1991

[B11] Molyneux DH Zagaria N Lymphatic filariasis elimination: progress in global programme development Ann Trop Med Parasitol 2002 96 Suppl 2 S15 40 12630391 10.1179/000349802125002374

[B12] Montresor A Crompton DWT Bundy DAP Hall A Savioli L Guidelines for the evaluation of soil-transmitted helminthiasis and schistosomiasis at community level 1998 Geneva WHO/CTD/SIP/98.91

[B13] World Health Organization Preventive chemotherapy in human helminthiasis: coordinated use of anthelminthic drugs in control interventions: a manual for health professionals and programme managers 2006 Geneva

[B14] Miguel E Kremer M Worms: Identifying Impacts on Education and Health in the Presence of Treatment Externalities Econometrica 2004 72 1 159 217 10.1111/j.1468-0262.2004.00481.x

[B15] Njenga SM Mwandawiro CS Wamae CN Mukoko DA Omar AA Shimada M Bockarie MJ Molyneux DH Sustained reduction in prevalence of lymphatic filariasis infection in spite of missed rounds of mass drug administration in an area under mosquito nets for malaria control Parasit Vectors 2011 4 1 90 10.1186/1756-3305-4-90 21612649 PMC3125382

[B16] Fox LM Furness BW Haser JK Desire D Brissau JM Milord MD Lafontant J Lammie PJ Beach MJ Tolerance and efficacy of combined diethylcarbamazine and albendazole for treatment of Wuchereria bancrofti and intestinal helminth infections in Haitian children Am J Trop Med Hyg 2005 73 1 115 121 16014845

[B17] Rajendran R Sunish IP Mani TR Munirathinam A Arunachalam N Satyanarayana K Dash AP Community-based study to assess the efficacy of DEC plus ALB against DEC alone on bancroftian filarial infection in endemic areas in Tamil Nadu, south India Trop Med Int Health 2006 11 6 851 861 10.1111/j.1365-3156.2006.01625.x 16772007

[B18] Knopp S Rinaldi L Khamis IS Stothard JR Rollinson D Maurelli MP Steinmann P Marti H Cringoli G Utzinger J A single FLOTAC is more sensitive than triplicate Kato-Katz for the diagnosis of low-intensity soil-transmitted helminth infections Trans R Soc Trop Med Hyg 2009 103 4 347 354 10.1016/j.trstmh.2008.11.013 19168197

[B19] Utzinger J Rinaldi L Lohourignon LK Rohner F Zimmermann MB Tschannen AB N'Goran E K Cringoli G FLOTAC: a new sensitive technique for the diagnosis of hookworm infections in humans Trans R Soc Trop Med Hyg 2008 102 1 84 90 10.1016/j.trstmh.2007.09.009 18028969

[B20] Bundy DA Chandiwana SK Homeida MM Yoon S Mott KE The epidemiological implications of a multiple-infection approach to the control of human helminth infections Trans R Soc Trop Med Hyg 1991 85 2 274 276 10.1016/0035-9203(91)90054-3 1887492

[B21] Barreto ML Loureiro S Melo AS Anjos CF The effect of Schistosoma mansoni infection on child morbidity in the State of Bahia, Brazil. II--Analysis at the individual level Rev Inst Med Trop Sao Paulo 1985 27 4 167 171 3938563 10.1590/s0036-46651985000400001

[B22] Crompton D Montresor A Nesheim M Savioli L eds Controlling disease due to helminth infections 2003 Geneva

[B23] Richards FO Jr Eigege A Miri ES Jinadu MY Hopkins DR Integration of mass drug administration programmes in Nigeria: The challenge of schistosomiasis Bull World Health Organ 2006 84 8 673 676 10.2471/BLT.06.029652 16917658 PMC2627425

